# Transcriptional profiles of non-neuronal and immune cells in mouse trigeminal ganglia

**DOI:** 10.3389/fpain.2023.1274811

**Published:** 2023-10-31

**Authors:** Jennifer Mecklenburg, Sergey A. Shein, Mostafa Malmir, Anahit H. Hovhannisyan, Korri Weldon, Yi Zou, Zhao Lai, Yu-Fang Jin, Shivani Ruparel, Alexei V. Tumanov, Armen N. Akopian

**Affiliations:** ^1^ Department of Endodontics, School of Dentistry, The University of Texas Health Science Center at San Antonio (UTHSCSA), San Antonio, TX, United States; ^2^ Microbiology, Immunology & Molecular Genetics Departments, School of Medicine, UTHSCSA, San Antonio, TX, United States; ^3^ Department of Electrical and Computer Engineering, the University of Texas at San Antonio, San Antonio, TX, United States; ^4^ Molecular Medicine, School of Medicine, UTHSCSA, San Antonio, TX, United States; ^5^ Greehey Children’s Cancer Research Institute, UTHSCSA, San Antonio, TX, United States

**Keywords:** trigeminal ganglia, glia, immune cells, stromal cells, macrophages, neutrophils, single-cell RNA-seq

## Abstract

Non-neuronal cells constitute 90%–95% of sensory ganglia. These cells, especially glial and immune cells, play critical roles in the modulation of sensory neurons. This study aimed to identify, profile, and summarize the types of trigeminal ganglion (TG) non-neuronal cells in naïve male mice using published and our own data generated by single-cell RNA sequencing, flow cytometry, and immunohistochemistry. TG has five types of non-neuronal cells, namely, glial, fibroblasts, smooth muscle, endothelial, and immune cells. There is an agreement among publications for glial, fibroblasts, smooth muscle, and endothelial cells. Based on gene profiles, glial cells were classified as myelinated and non-myelinated Schwann cells and satellite glial cells. *Mpz* has dominant expression in Schwann cells, and *Fabp7* is specific for SCG. Two types of Col1a2^+^ fibroblasts located throughout TG were distinguished. TG smooth muscle and endothelial cells in the blood vessels were detected using well-defined markers. Our study reported three types of macrophages (Mph) and four types of neutrophils (Neu) in TG. Mph were located in the neuronal bodies and nerve fibers and were sub-grouped by unique transcriptomic profiles with *Ccr2*, *Cx3cr1*, and *Iba1* as markers. A comparison of databases showed that type 1 Mph is similar to choroid plexus-low (CP^lo^) border-associated Mph (BAMs). Type 2 Mph has the highest prediction score with CP^hi^ BAMs, while type 3 Mph is distinct. S100a8^+^ Neu were located in the dura surrounding TG and were sub-grouped by clustering and expressions of *Csf3r*, *Ly6G*, *Ngp*, *Elane*, and *Mpo*. Integrative analysis of published datasets indicated that Neu-1, Neu-2, and Neu-3 are similar to the brain Neu-1 group, while Neu-4 has a resemblance to the monocyte-derived cells. Overall, the generated and summarized datasets on non-neuronal TG cells showed a unique composition of myeloid cell types in TG and could provide essential and fundamental information for studies on cell plasticity, interactomic networks between neurons and non-neuronal cells, and function during a variety of pain conditions in the head and neck regions.

## Introduction

Multiple reports reported that dorsal root ganglion (DRG) glial (1–4) and immune cells, especially macrophages (Mph) and neutrophils (Neu), play critical roles in nociceptive signal transmission (5–8). Studies suggested that ganglion non-neuronal cells are capable of sensitizing neurons (5, 2, 3) by directly communicating with them and changing their gating properties (7, 9, 4). Accordingly, information on transcriptional profiles for non-neuronal sensory ganglion cells is critically important for examining intercellular signal transduction among neuronal and non-neuronal cells. Such fundamental information could also be used to explain a variety of mechanisms during interactions of sensory neuron soma with non-neuronal cells within the ganglia. For example, the information has been used to establish interactomic networks between sensory neurons and non-neuronal cells (10). In addition, it could constitute a baseline in the investigation of trigeminal ganglion (TG) non-neuronal cell plasticity in different pain models and conditions for the head and neck regions. Single-nucleus RNA sequencing (snRNA-seq) of DRG and TG cells has previously generated transcriptomic profiles for both sensory neurons and non-neuronal ganglion cells (11–14). Single-cell RNA sequencing (scRNA-seq) studies are complex and often produce variable outcomes. These outcomes depend on several factors such as ganglial type (DRG vs. TG vs. nodose ganglia), snRNA-seq vs. scRNA-seq, nucleus/cell isolation approach, sequencing depth, and clustering analysis (11–14). Hence, every independent study contributes novel information and refines the previously reported data. Thus, seven non-neuronal subtypes, namely, satellite glial cells (SGC), myelinating and non-myelinating Schwann cells, Mgp^+^ and Dcn^+^ fibroblasts, immune cells, and vascular endothelial cells, were identified in TG using snRNA-seq (14), wherein nuclei were isolated using a density gradient method (12). Other snRNA-seq and scRNA-seq studies reported eight (15) or five (13) types of non-neuronal cells in TG, namely, SGC, myelinating and non-myelinating Schwann cells, fibroblasts, myofibroblasts, immune cells, and vascular endothelial cells. snRNA-seq of DRG cells revealed nine types of non-neuronal cells, namely, SGC, myelinating and non-myelinating Schwann cells, one group of fibroblasts, pericytes, and vascular endothelial cells, and three types of immune cells, i.e., Mph, B cells, and Neu (12).

Multiple studies on the roles of ganglion glial cells in the regulation of sensory neurons require specific markers to distinguish SGC from Schwann cells and other non-neuronal cells. It is not entirely clear whether such markers exist. In addition, the function of ganglion fibroblasts is largely unknown. In this respect, more information on their gene profiles and locations within TG is needed. Studies reported three immune cell types in DRG (12) and only one group in TG (13–15). Furthermore, additional studies regarding this topic could be valuable. Accordingly, this study aimed to identify, profile, and summarize TG non-neuronal cell types in naïve male mice using published and our own data generated by scRNA-seq, flow cytometry, and immunohistochemistry (IHC).

## Materials and methods

### Ethical approval and mouse lines

The reporting in the manuscript follows the recommendations in the ARRIVE guidelines [*PLoS Bio*. (2010) **8**(6): e1000412]. We also followed the guidelines issued by the National Institutes of Health (NIH) and the Society for Neuroscience (SfN) in minimizing the number of animals used and their suffering. All animal experiments conformed to the protocols approved by the University Texas Health Science Center at San Antonio (UTHSCSA) Institutional Animal Care and Use Committee (IACUC). The protocol numbers are 20190114AR and 20220069AR.

Experiments were performed on the following male mice: 10–18-week-old C57BL/6 wild type (WT); Col1a2-cre-ER (stock no: 029567); tdTomato (aka Ai14; stock no: 007914); and Ccr2^RFP^/Cx3cr1^GFP^ (stock no: 032127) on the B6.129 background. All mouse lines were purchased from the Jackson Laboratory (Bar Harbor, ME, USA) and were bred in the UTHSCSA LAR facilities.

### TG isolation and single-cell preparation

There are several approaches to dissecting TG tissues. One of them is to collect the TG with surrounding dura and another is to isolate the dura-free TG. We have isolated TG with surrounding dura for scRNA-seq, TG without dura for IHC, and both preparations of TG with and without dura for flow cytometry. Briefly, prior to TG dissections, the animals were perfused with cold phosphate-buffered solution (PBS) to eliminate the contributions of immune cells from blood to the scRNA-seq and flow cytometry data. For IHC, mice were perfused with 4% paraformaldehyde prior to tissue dissections. TG were dissected from the skull base after the removal of the brain. For dura-free TG, V1–V3 were cut close to TG, and then dura-free TG was lifted by a spatula. For TG with dura, continuous cuts were made all around TG, resulting in a dissected TG covered by dura.

Mice were perfused with PBS to flash out the blood cells from the tissues, including TG. Dissected TG were collected in ice-cold HBSS buffer and subjected to preparation of single-cell suspension for scRNA-seq or flow cytometry. Single-cell suspension was generated using Liberase and Dispase II as described previously (16). After this step, single-cell suspension was processed in two different ways. For the first scRNA-seq experiment, fractions enriched with sensory neurons were obtained using the Percoll gradient as described previously (16). For the second scRNA-seq experiment, viable TG cells were purified by flow cytometry using Calcein Violet-AM/Helix NP NIR (BioLegend) dual live/dead stain. Calcein Violet-AM is a cell-permeable fluorescent probe cleaved and activated in live cells by esterases, whereas Helix NP NIR is impermeable to live cells and detects the nucleic acids of dead cells. Briefly, TG cells were stained first with 0.1 μM of Calcein Violet-AM for 40 min at room temperature, followed by 5 nM of Helix NP NIR. Calcein Violet-AM^+^/Helix NP NIR-gated TG cells were sorted to DMEM/5% fetal calf serum (FCS) medium using the BD FACSAria Fusion cell sorter equipped with a 100 μm nozzle. Sorted cells were centrifuged and resuspended in 15 μl of 1X PBS, 0.04% BSA, and 0.1 U/μl RNase inhibitor. For flow cytometry experiments, single-cell suspensions after the Liberase–Dispase step (see above) were stained with a panel of antibodies as described below.

### scRNA-seq procedures, clustering, visualization, and annotation

**Figure 1:**
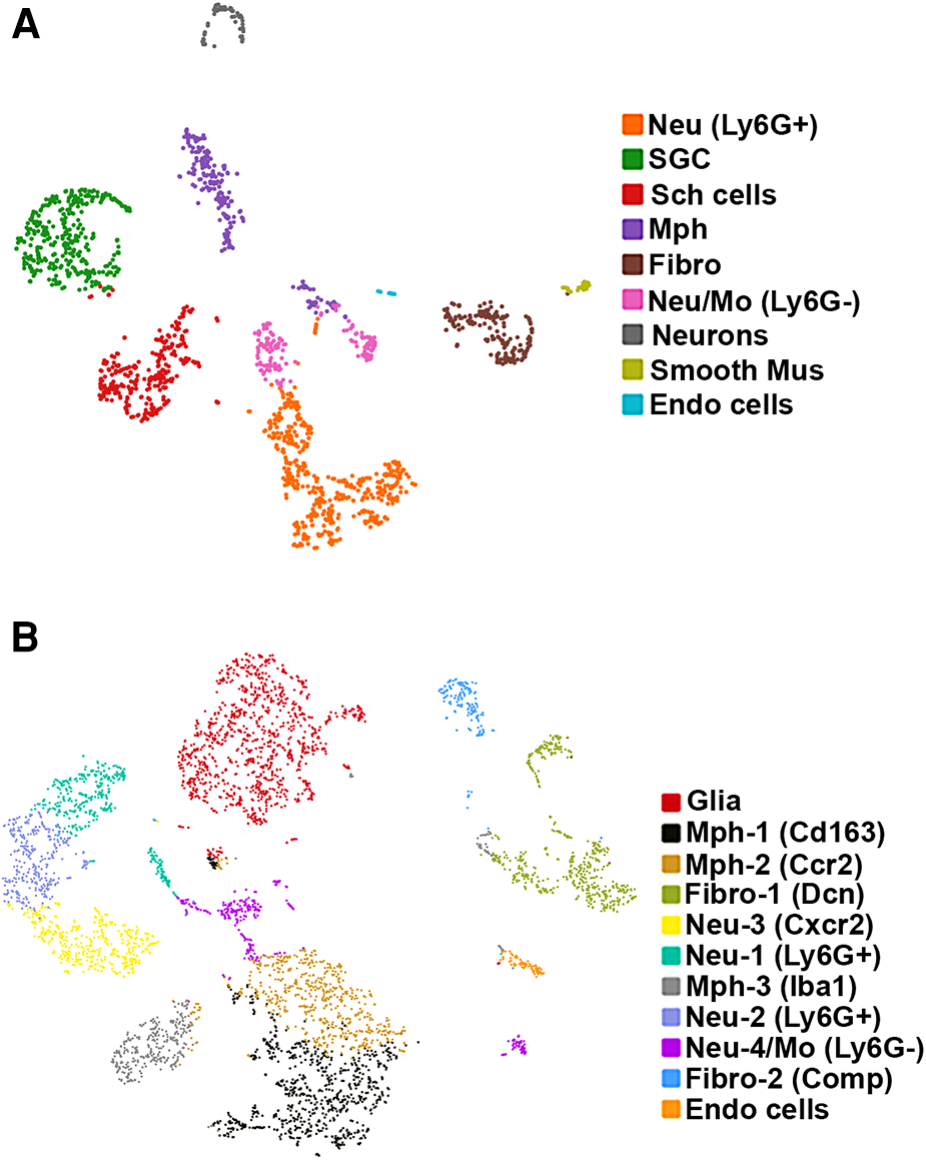
scRNA-seq of mouse TG non-neuronal cells. Two independent replicates were performed. (**A**) tSNE plot of scRNA-seq data from experiment 1 from 2,859 live FACS-isolated TG cells from three mice. Cell types are indicated and are represented by different colors. *Nue* (Ly6G+) are Ly6G-positive neutrophils (Neu); SGC, satellite glial cells; Sch cells, Schwann cells; Mph, macrophages; Fibro, fibroblasts; Neu/Mo (Ly6G-), monocyte-derived Neu-like Ly6G-negative cells; Smooth Mus, blood vessel smooth muscle cells; and Endo cells, endothelial cells. (**B**) tSNE plot of scRNA-seq data from experiment 2 from 6,362 live FACS-isolated TG cells from three mice. Cell types are indicated and are represented by different colors. Glia are a combination of satellite glial cells and Schwann cells; Mph-1 (Cd163), type 1 macrophage with Cd163 as a marker; Mph-2 (Ccr2), type 2 macrophage with Ccr2 as a marker; Fibro-1 (Dcn), type 1 fibroblasts with Dcn as a marker; Neu-3 (Cxcr2), type 3 Neu with Cxcr2 as a marker; Neu-1 (Ly6G+), Ly6G-positive type 1 Neu; Mph-3 (Iba1), type 2 macrophage with Iba1 as a marker; Neu-2 (Ly6G+), Ly6G-positive type 2 Neu; Neu-4/Mo (Ly6G−), Ly6G-negative monocyte-derived Neu-like type 4 cells; Fibro-2 (Comp), type 1 fibroblasts with Comp as a marker; and Endo cells, endothelial cells.

TG from three mice were used to generate single-cell suspension for one experiment. The experiment was performed in two independent replicates: the first preparation used the Percoll step (16) and the second used the Calcein Violet-AM/Helix NP Blue sorting approach (see previous sub-section of Materials and methods section). All clustering methods, visualization (tSNE, UMAP), and analysis were done by the 10X tool loupe browser (https://support.10xgenomics.com/single-cell-gene-expression/software/visualization/llatest/what-is-loupe-cell-browser). The 10X single-cell raw sequencing data from both next generation sequencing runs were processed following the 10X single-cell gene expression pipeline (https://www.10xgenomics.com/support/single-cell-gene-expression). The 10X software Cell-Ranger mkfastq was used for base-calling and generating raw fastqs. Cell-Ranger count was used to align the fastq reads to the reference transcriptome (refdata-gex-mm10-2020-A) and to generate a gene count matrix and a “cloupe” file. In addition, the “cloupe file” was put in the “Loupe Browser” for data visualization and preliminary analysis. Cells with >500 unique genes, <15,000 total UMIs, and <10% of the counts deriving from mitochondrial genes were included for analysis. We used two similar different clustering approaches. For the first scRNA-seq experiment, K-means was employed (Figure 1A), while for the second scRNA-seq experiment, a graph-based approach was used for analysis (Figure 1B). For both runs, we employed tSNE plots for visualization. Commonly, doublet or low-quality clusters could significantly be enriched for at least four mitochondrial genes [fold change (FC) > 2, false discovery rate (*P*_adj_) < 0.05)] have no enriched cluster marker genes (FC > 5, *P*_adj_ < 0.05). We did not have such clusters in the final presented data.

### Comparisons of TG immune cell transcriptional profiles with published datasets

The label transfer method provided by the Seurat package was used to project labels from a reference dataset to a query dataset by identifying shared “anchors” between two datasets (17). The identified anchors were used to integrate these two datasets as a batch-corrected expression matrix for all cells, enabling them to be jointly analyzed for cell-type clustering. Label transfer has been widely adopted in scRNA-seq studies by leveraging rich annotations from reference datasets to interpret and understand the cellular composition and state of query datasets (18). In this study, the reference dataset contains 21,966 CD45^+^ cells extracted from the entirety and border areas of male C57BL/6 mice at 9 weeks of age, which have been labeled as 14 distinct cell types (19). Specifically, the reference data have 10,947 Mph sourced from both the entire brain and its bordering regions containing seven cell types, among which six were unique subtypes of border-associated Mph (BAMs) cells and one microglial cell type. Annotation of these cells and the gene expressions (GSE128855) can be downloaded from (20) (https://www.brainimmuneatlas.org/). The label transfer algorithm was then applied to interpret our own dataset for cell composition.

### Flow cytometry

Flow cytometry was used to assess immune cell profiles in TG. Single-cell suspensions were first stained for viability using Zombie NIR™ fixable viability kit (BioLegend, San Diego, CA, USA) for 20 min on ice in PBS at pH 7.2 combined with FcR blocking antibody (1 μg, clone 2.4G2; Bio X Cell, Lebanon, NH, USA) to block non-specific binding. Cells then were washed with 2% FBS/PBS and stained with antibodies against surface antigens for 30 min on ice. Fluorochrome-conjugated antibodies against mouse CD45 (clone 30-F11), CD3 (clone 17A2), B220 (clone RA3-6B2), CD11b (clone M1/70), CD64 (clone X54-5/7.1), CD11c (clone N418), NK1.1 (clone PK136), MHC-II (clone 2G9), Ly-6G (clone 1A8), Ly-6C (clone KH1.4), CCR2 (clone 475301), CX3CR1 (clone SA011F11), and Siglec-F (clone S17007l) were purchased from BioLegend (San Diego, CA, USA) or BD Biosciences (San Jose, CA, USA). Flow cytometry was performed using Aurora (Cytek Biosciences, CA, USA). Data were analyzed using FlowJo LLC v10.6.1 software.

**Figure 2:**
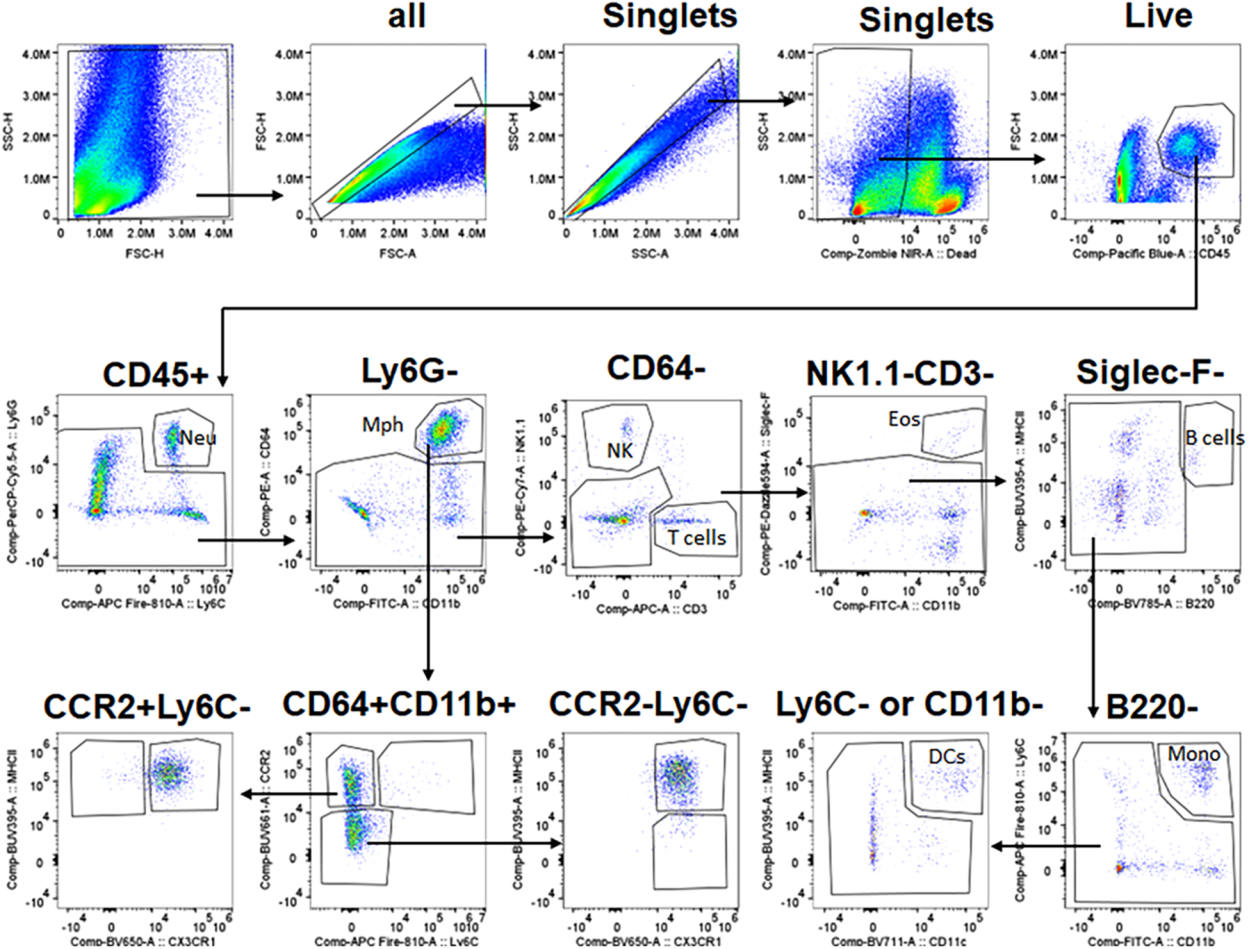
Gating strategy for flow cytometry. The panel shows the sequence of gating events. The first step is the selection of live singlets and then the separation of CD45^+^ cells. CD45^+^ cells are gated into different types of immune cells using sets of specific markers. Immune cell types and markers are indicated. The sequence of gaining events is marked by arrows. Neu, neutrophils; Mph, macrophages; NK, natural killer cells; Eos, eosinophils; DCs, dendritic cells; Mono, monocytes.

The gating strategy to select immune populations in TG is shown in Figure 2. Briefly, live/singlets/CD45^+^ cells were gated using the markers listed below to define specific cell populations: monocytes (Mo, Ly6G^−^/Siglec-F^−^/NK1.1^−^/Ly6C^+^/CD11b^−^); total Mph (CD64^high^/CD11b^+^); type 1 macrophage (Mph-1, Ly6C^+^/CCR2^+^); type 2 macrophage (Mph-2, Ly6C^−^/CCR2^+^/CX3CR1^+^/MHCII^+^); type 3 macrophage (Mph-3, Ly6C^−^/CCR2^+^/CX3CR1^−^/MHCII^+^); type 4 macrophage (Mph-4, Ly6C^−^/CCR2^−^/CX3CR1^+^/MHCII^+^); type 5 macrophage (Mph-5, Ly6C^−^/CCR2^−^/CX3CR1^+^/MHCII^−^) Ly6G^+^ Neu (Nph, CD11b^+^/Ly6G^+^/Ly6C^+^); dendritic cells (DCs, B220^−^/CD64^−^/Ly6C^−^/MHCIIhigh/CD11c^+^); eosinophils (Eos; Siglec-F^+^/CD11b^+^/CD64^−^); natural killer cells (NK, NK1.1^+^/CD3^−^); B cells (B, B220^+^/MHCII^+^); and T cells (T, CD3^+^/NK1.1^−^).

### Immunohistochemistry

For IHC, we used naïve WT and Ccr2^RFP^Cx3cr1^GFP^ and Col1a2^cre^/Ai14^fl/−^ reporter male mice. TG tissues from 4% paraformaldehyde perfused mice were isolated, post-fixed with 4% paraformaldehyde for 1 h, cryoprotected with 10% and then 30% sucrose in phosphate buffer overnight, embedded in Neg-50 (Richard–Allan Scientific, Kalamazoo, MI, USA), and cryo-sectioned at 25–30 μm. IHC was carried out as previously described (21). The following antibodies were used: anti-Iba1 rabbit polyclonal (Thermo Fisher Scientific, San Diego, CA, USA; Cat: PA5-27436; 1:300); anti-s100a8 rat IgG2B monoclonal (R&D Systems; clone # 335806; Cat: MAB3059-SP 1:200); and anti-NFH chicken polyclonal (Novus Biologicals; Cat: NB300-217; 1:2000). Donkey Alexa Fluor secondary antibodies were obtained from Jackson ImmunoResearch (1:200; West Grove, PA, USA). Control IHC was performed on tissue sections processed as described but either lacking primary antibodies or lacking primary and secondary antibodies. The images were acquired using a Keyence BZ-X810 All-in-One Fluorescence Microscope (Keyence, Itasca, IL, USA). The gain setting was constant during acquisition, and it was established on no primary control slides. All Images taken were z-stack images and were processed with the Adobe Photoshop CS2 software. Cells on microphotography were counted within the entire field captured with a ×20 objective. The field of view is approximately 350 μm  × 350 μm for our microscope equipped with a ×20 objective.

### Statistical analysis

The GraphPad Prism 8.0 (GraphPad, La Jolla, CA, USA) was used for all statistical analyses of data related to non-scRNA-seq. Data in the figures were expressed as mean ± standard error of the mean (SEM), with “*n*” referring to the number of animals per group. The differences between groups with one variable were assessed by chi-square analysis with Fisher’s exact test, unpaired *t*-test, or regular 1-way ANOVA with Bonferroni’s *post-hoc* tests, and each column was compared to all other columns. A difference was accepted as statistically significant when *p* < 0.05. Interaction *F* ratios and the associated *p* values were also reported.

## Results

### scRNA-seq of TG non-neuronal cells

We carried out two replicates for scRNA-seq on 12 left and right TG from six naïve, WT C57BL/6 male mice. Bilateral TG from three mice were combined per replicate. For the first experiment, single-cell suspension preparation had the Percoll step (see Materials and methods section). For the second experiment, single-cell suspension preparation had two cycles of FACS sorting step for isolation of viable cells (see Materials and methods section). For the first replicate experiment, 2,859 TG cells were present in scRNA-seq, and an average of 1,211 genes were detected per cell. For the second replicate experiment, 6,362 TG cells were present in scRNA-seq, and an average of 1,580 genes were detected per cell. All TG cells in each experiment were clustered together. The Loupe Browser developed by 10X Genomics was used for data visualization (12, 13). We clustered and classified cell types using K-means for both replicates (22). We also used graph-based clustering for the second replicate (23). K-means and graph-based clustering for the second replicate produced identical results. Clusters enriched with mitochondrial genes were excluded. Clusters enriched with the expression of *Pirt* were assigned as sensory neuronal clusters, and the remaining clusters were classified as non-neuronal cells (24, 25).

We detected nine clusters (Cluster-1 has 471 cells, Cluster-2 with 351, Cluster-3 with 288, Cluster-4 with 239, Cluster-5 with 192, Cluster-6 with 168, Cluster-7 with 72, Cluster-8 with 37, and Cluster-9 with 21 cells) in the first experimental run/replicate (Figure 1A) and 11 clusters (Cluster-1 has 1,126 cells, Cluster-2 with 815, Cluster-3 with 618, Cluster-4 with 561, Cluster-5 with 446, Cluster-6 with 382, Cluster-7 with 356, Cluster-8 with 333, Cluster-9 with 311, Cluster-10 with 185, and Cluster-11 with 81 cells) in the second run/replicate (Figure 1B). Cell types were identified according to the significant enrichment of markers (*P*_adj_ < 0.05; FC > 5) and also by specificity in expression for certain genes (FC > 10). It could be noted that a neuronal cluster was not detected in the second experimental trial. This could be explained by the no-neuron Percoll enriching step in the second trial. This also indicates that scRNA-seq has a substantial preference for sequencing non-neuronal cells over sensory neurons (16, 26).

### Glial cells in TG

**Figure 3:**
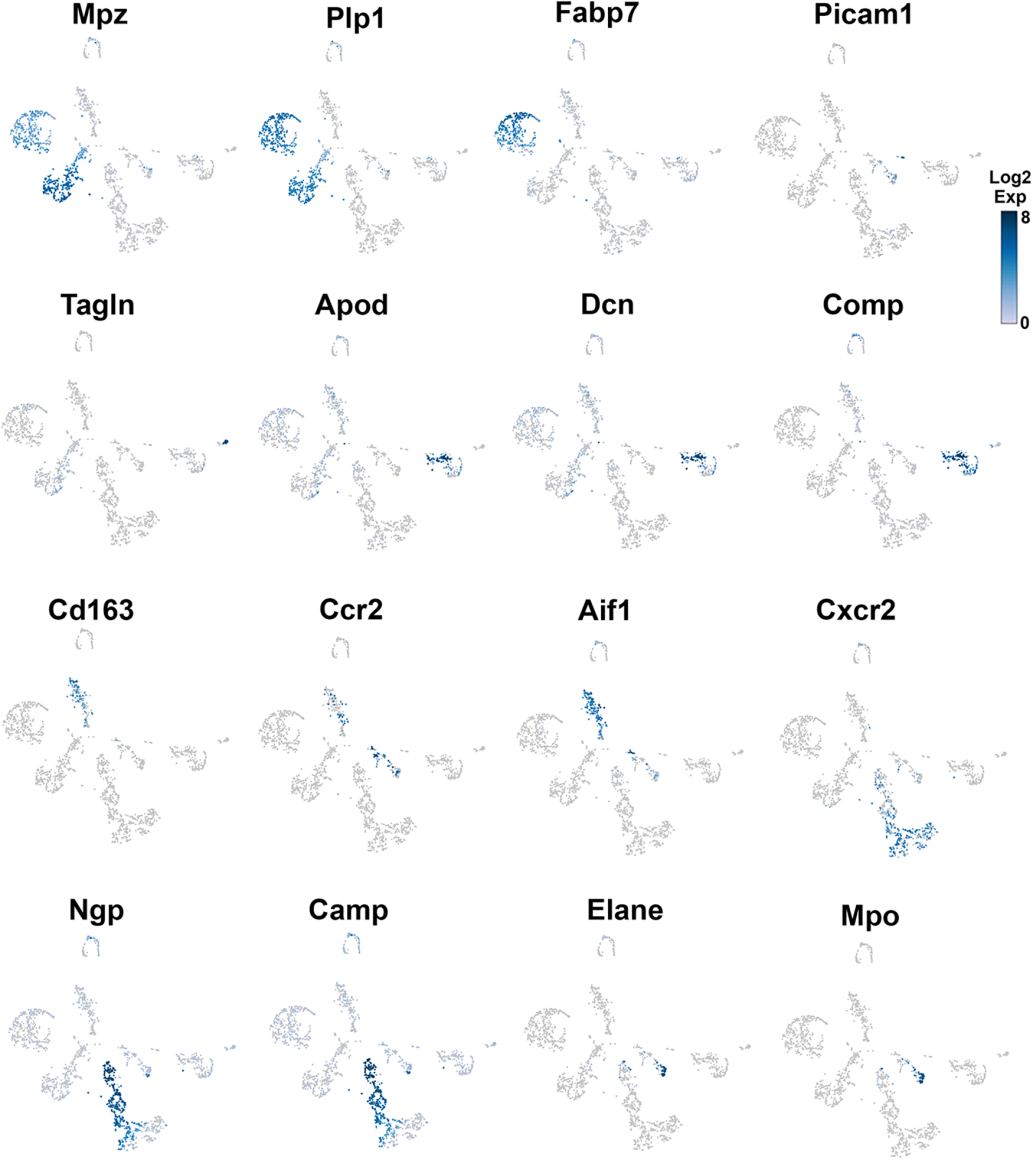
Expression of markers in TG non-neuronal cells (experiment 1). The expressions of select cell-type marker genes for TG non-neuronal cells clustered in experiment 1 are displayed in individual cells and within clusters. The marker genes are indicated.

Glial cell sub-types were revealed after analysis of the first trial (Figure 1A), while the second trial and analysis revealed only a single glial group (Figure 1B). Schwann cells were recognized by an enrichment with *Mpz* (Figures 1A, 3 and Table 1). SGC cells were distinguished by a specific marker—*Fabp7* (Figures 1A, 3 and Table 1). *Fabp7* as an SGC marker was also noted in previous reports (27, 15). *Apoe*, suggested as an SGC marker, was found to also be expressed at high levels in Mph in our study (Table 1) (14, 15). *Plp1* is also not suitable as an SGC marker, since it is presented at an equal level in both Schwann cells and SGC (Figures 1A, 3 and Table 1) (28–30). Previous publications distinguished myelinating and non-myelinating Schwann cells using *Ncmap*, *Prx*, *Scn7a*, and *Cdh19* (14, 15). The clustering of our data did not reveal a distinct myelinating Schwann cell subset. However, *Ncmap^+^* and *Prx^+^* cells were encountered among Schwann cells and were absent in SGC, while *Cdh19^+^* and *Scn7a*^+^ cells were present only among SGC (see the Supplementary Material). Despite glial cells containing distinct markers, it could be noted that low-to-moderate levels of expression of these genes have been reported in sensory neurons (31, 13, 14). Overall, our scRNA-seq data have shown that TG glial cells are divided into two groups, namely, Schwann cells and SGC, which have distinct transcriptional profiles. Previous publications showed both Schwann cells and SGC could be clustered into a variety of subtypes. Thus, Schwann cells were classified into myelinating and non-myelinating, whereas SGC types were divided into general resident, sensory, immediate early gene (IEG), and immune-responsive groups (32, 14, 15).

**Table 1:** Markers for non-neuronal cells in TG.

ID	SchC	SGC	Mus	Endo	Fibro-1	Fibro-2	Mph1	Mph2	Mph3	Neu1	Neu2	Neu3	Neu4
Mpz	152	8	–	–	–	–	–	–	–	–	–	–	–
Mbp	27	1.8	–	–	–	–	–	–	–	–	–	–	–
Plp1	13	15	–	–	–	–	–	–	–	–	–	–	–
Apoe	1.6	188	1.6	–	27	–	274	207	217	–	–	–	–
Fabp7	–	26	–	–	–	–	–	–	–	–	–	–	–
Tie	–	–	–	1	–	–	–	–	–	–	–	–	–
Pecam1	–	–	–	2.7	–	–	–	–	–	–	–	–	–
Tagln	–	–	46	–	–	–	–	–	–	–	–	–	–
Tpm1	–	–	18	–	–	–	–	–	–	–	–	–	–
Myh11	–	–	17	–	–	–	–	–	–	–	–	–	–
Col1a2	–	–	–	–	7.7	13	–	–	–	–	–	–	–
Apod	–	–	–	–	293	9	–	–	30	–	–	–	–
Dcn	–	–	–	–	174	22	–	–	9	–	–	–	–
Vtn	–	–	–	–	3.6	–	–	–	–	–	–	–	–
Ccl11	–	–	–	–	7.8	–	–	–	–	–	–	–	–
Mgp	–	–	8.5	8.6	16	117	–	–	6	–	–	–	–
Comp	–	–	–	–	–	5.3	–	–	–	–	–	–	–
IL33	–	–	–	–	2.7	0.5	–	–	–	–	–	–	–
Pdgfra	–	–	–	–	1.1	–	–	–	–	–	–	–	–
Pdgfrb	–	–	–	–	1.1	–	–	–	–	–	–	–	–
Csf1r	–	–	–	–	–	–	6	4	4	–	–	–	–
Ccr2	–	–	–	–	–	–	–	1.2	–	–	–	–	2.1
Cx3cr1	–	–	–	–	–	–	3.5	2.5	0.8	–	–	–	–
Cd68	–	–	–	–	–	–	2.5	2.3	2.1	–	–	–	–
Cd163	–	–	–	–	–	–	1.8	0.5	0.8	–	–	–	–
Aif	–	–	–	–	–	–	3.3	3.6	6	–	–	–	–
Cxcl9	–	–	–	–	–	–	–	–	1.4	–	–	–	–
Csf3r	–	–	–	–	–	–	–	–	–	4.3	–	0.8	–
Cxcr2	–	–	–	–	–	–	–	–	–	2.8	–	1.5	–
Cxcr4	–	–	–	–	–	–	–	–	–	2.6	–	0.9	–
Cxcl2	–	–	–	–	–	–	2.3	10	–	36	–	4	3
S100a8	–	–	–	–	–	–	–	–	–	319	590	615	16
S100a9	–	–	–	–	–	–	–	–	–	462	858	914	20
Ngp	–	–	–	–	1.3	–	–	–	–	–	240	99	2.3
Ly6G	–	–	–	–	–	–	–	0.9	–	–	4.8	4	–
Cd177	–	–	–	–	–	–	–	–	–	–	2.7	2.7	–
Camp	–	–	–	–	–	–	–	–	–	–	204	56	3
Ltf	–	–	–	–	–	–	–	–	–	–	40	19	–
Elane	–	–	–	–	–	–	–	–	–	–	2	–	5.5
Mpo	–	–	–	–	–	–	–	–	–	–	–	–	2.6
Pycard	–	–	–	–	–	–	–	–	–	–	–	–	1.25
Ramp1	–	–	3	–	–	–	–	–	–	–	–	–	1.1
Ccl5	–	–	–	–	–	–	–	–	–	–	–	–	3
Cd74	–	–	–	–	1.4	1.8	90	118	–	59	19	–	–
Ccl2	–	–	–	–	2.8	–	2.5	3.2	–	–	–	–	–
Itgam	–	–	–	–	–	–	*0.6*	*0.4*	*0.35*	1.2	1.3	1.7	0.5
Ptprc	–	–	–	–	–	–	1.6	1.4	0.6	5.9	1.3	2.7	2.4
Lyz2	–	–	–	–	–	–	66	69	54	30	77	56	63
Itgax	–	–	–	–	–	–	–	–	–	–	–	–	–
Cd3d	–	–	–	–	–	–	–	–	–	–	–	–	–
Cd22	–	–	–	–	–	–	–	–	–	–	–	–	–
Il2rb	–	–	–	–	–	–	–	–	–	–	–	–	–

SchC, Schwann cells; SGC, satellite glial cells; Mus, smooth muscle; Endo, endothelial cells; Fibro-1, Apod^+^/Dcn^high^ fibroblasts; Fibro-2, Comp^+^/Mgp^high^ fibroblasts; Mph1, CD163^high^ macrophages (Mph); Mph2, Cx3cr1^+^ Mph; Mph3, Aif1^high^ Mph; Neu-1, Cxcr2^high^/Cxcr4^+^ neutrophils (Neu); Neu-2, Ly6G^+^ Neu; Neu-3, Ly6G^+^ Neu; Neu4, Mpo^+^/Elane^high^/Ly6G^−^ Neu.

The – sign means an expression *lesser than 0.5*.

### Fibroblasts, smooth muscle cells, and endothelial cells in TG

**Figure 4:**
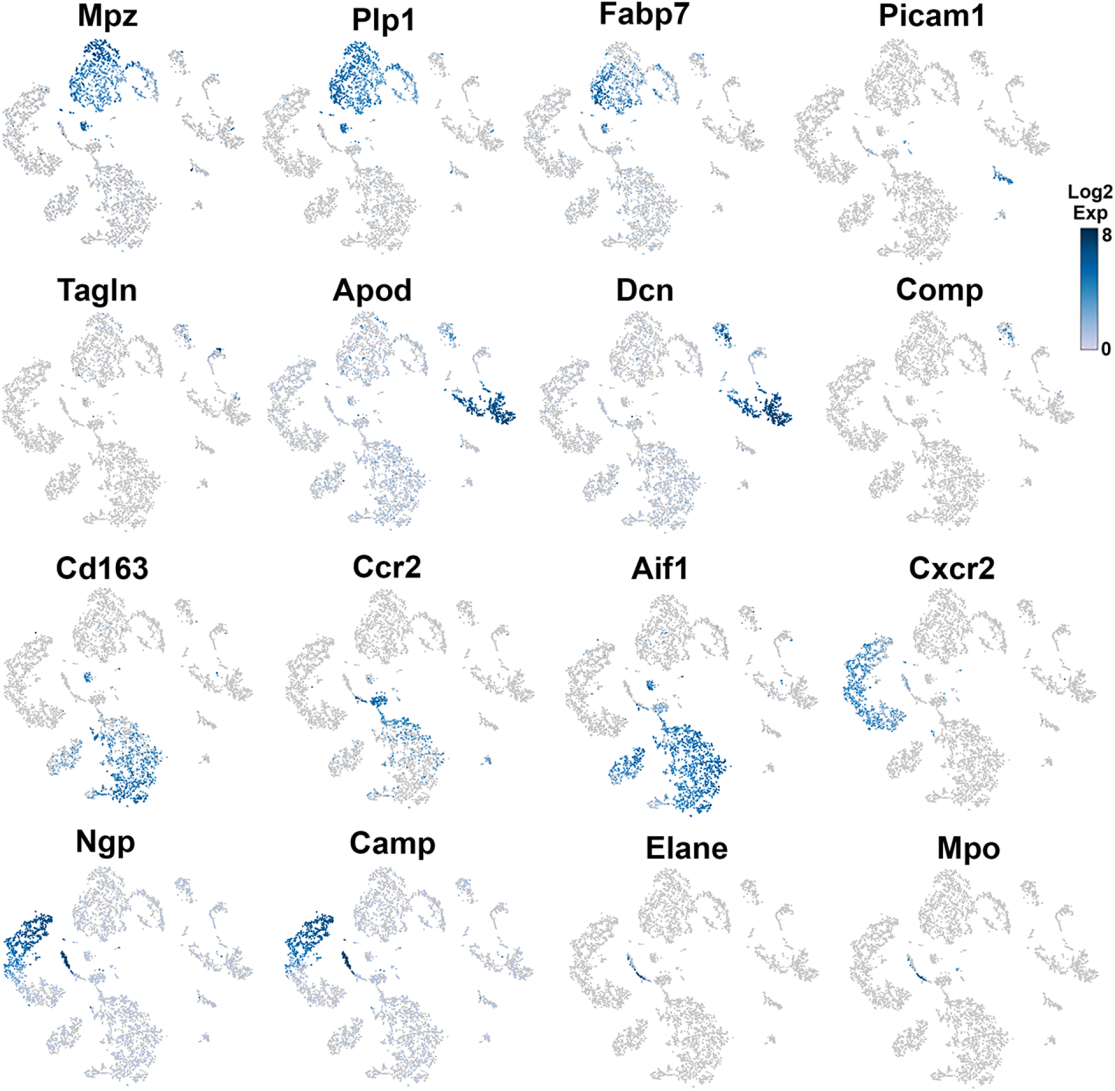
Expression of markers in TG non-neuronal cells (experiment 2). The expressions of select cell-type marker genes for TG non-neuronal cells clustered in experiment 2 are displayed in individual cells and within clusters. The marker genes are indicated.

Different types of fibroblasts were categorized after analysis of the second trial (Figure 1B), but not in the first trial (Figure 1A). Fibroblasts identified as Col1a2^+^ cells were represented by two distinct clusters (Figure 1B). The larger fibroblast subgroup, labeled as Fibro (Dcn), was *Apod^+^/Dcn^high^* and was distinguished by a combination of an enriched marker *Dcn* and a specific marker *Apod* (Figures 1A, 4 and Table 1). Unlike *Dcn*, *Apod* is expressed by many sensory neurons (31, 13, 14). The second subgroup, labeled as Fibro (Comp), was *Comp^+^/Mgp^high^*, was enriched with *Mgp*, contained a specific marker (*Comp*) (Figures 1A, 4 and Table 1), and was previously reported as myofibroblasts (15).

We and others also identified a small number of cells representing either smooth muscle or endothelial vascular cells (Figures 1A,B) (14, 15). Smooth muscle vascular cells contained a specific marker *Tagln*, and endothelial vascular cells were defined by a standard *Pecam1/CD31* marker (Figures 1A,B, 3, 4 and Table 1). In summary, there is an agreement that TG has two types of fibroblasts (i.e., Apod^+^/Dcn^high^ and Comp^+^/Mgp^high^) and vascular smooth muscle and endothelial cells (13–15).

### Immune cells in TG

Immune cells in TG were reported as one single group (13–15), though DRG immune cells were split into three groups, namely, Mph, Neu, and B cells (12). Our first trial revealed a group of Mph and two groups of Neu (Figure 1A). The second trial, which was not enriched with sensory neurons, contained more cells and deeper reads, and we split these groups into three Mph groups and four Neu groups (Figure 1B). Mph were recognized by the expression of CD64 (aka *Fcgr1*). The first group of Mph was enriched with *Cd163*, a marker for M2-type Mph (33, 34), but contained other M1-type Mph markers, such as *Csfr1*, *Cx3cr1*, *Cxcr1*, and *Cd68* (Figures 1B, 4 and Table 1). The second group of Mph had a relatively specific marker, *Ccr2*, and was also expressing *Csfr1*, *Cxcr1*, and *Cd68* (Figures 1B, 4 and Table 1). The third group of Mph was classified as *Aif1*^high^ (aka Iba1), which also specifically expressed *Cxcl9* (Figures 1B, 4 and Table 1). This group had a low level of *Cd163*, had no *Ccr2*, and expressed several fibroblast markers (Figure 4, Table 1).

Neu were recognized by the expression of *s100a8* and *s100a9* (35, 36). Four Neu groups were split into two domains, i.e., Ly6G^−^ and Ly6G^+^ (Table 1). The Ly6G^+^ Neu groups (Neu-2 and Neu-3) were similar and had high levels of *Cd177*, *Camp*, and *Ngp* expressions (Table 1). Nevertheless, Neu-2 and Neu-3 were differentiated by transcriptional profiles and expressions of *Elane* in Neu-2 and *Cxcl2* and *Cxcr2* in Neu-3 (Figures 1B, 3, 4 and Table 1). The Ly6G^−^ Neu groups (Neu-1 and Neu-4) were substantially different from Ly6G^+^ Neu and were dissimilar to each other (Figure 1B, Table 1). Thus, Neu-1 was enriched with *Cxcr4* and *Csf3r*, while Neu-4 was dominated with *Ccl5*, *Elane*, *Pycard*, and *Mpo* (Figures 3, 4 and Table 1).

Next, we looked at expressions of standard immune cell markers in clusters of TG non-neuronal cells. A pan-immune cell marker, *Ptprc* (aka CD45), was expressed by all TG immune cells. However, the expression level was low (Table 1). A pan-myeloid cell marker *Itgam* (aka CD11b) was presented at unusually low levels in Mph and at low-to-moderate levels in Neu (Table 1). A Mph and monocyte marker, *Ccl2*, was expressed by a subset of TG Mph and *Apod^+^/Dcn^high^* fibroblasts (Table 1). A myeloid cell marker, *CD74*, was TG Mph and Neu subsets and at lower levels in TG fibroblasts (Table 1). We found that the most suitable and highly expressed marker for all TG immune cells was *Lyz2* (aka LyzM; Table 1). scRNA-seq showed that the markers for other immune cell types, such as *Itgax* (aka CD11c) for DCs, *Il2rb* (aka CD122) for natural killer cells (NK), *Cd3d* and *Cd3e* for T cells, and *Cd22* for B cells, are not present in TG (Table 1). Altogether, TG from naïve mice had exclusively a subset of myeloid cells, which could be sub-grouped into several Mph and Neu types with distinct transcriptional profiles.

### Visualizations of non-neuronal cells in TG by IHC

Previous publications thoroughly characterized locations of different glial cell types, including distinct SGC groups, in TG and DRG, using *in situ* and IHC (28, 32). Mph and Neu were mainly characterized and visualized in DRG and were often presented as a single group (37–41). Here, we investigated whether several groups of Mph, Neu, and fibroblasts could be detected in TG. To do so, we used two approaches, i.e., IHC and flow cytometry.

**Figure 5:**
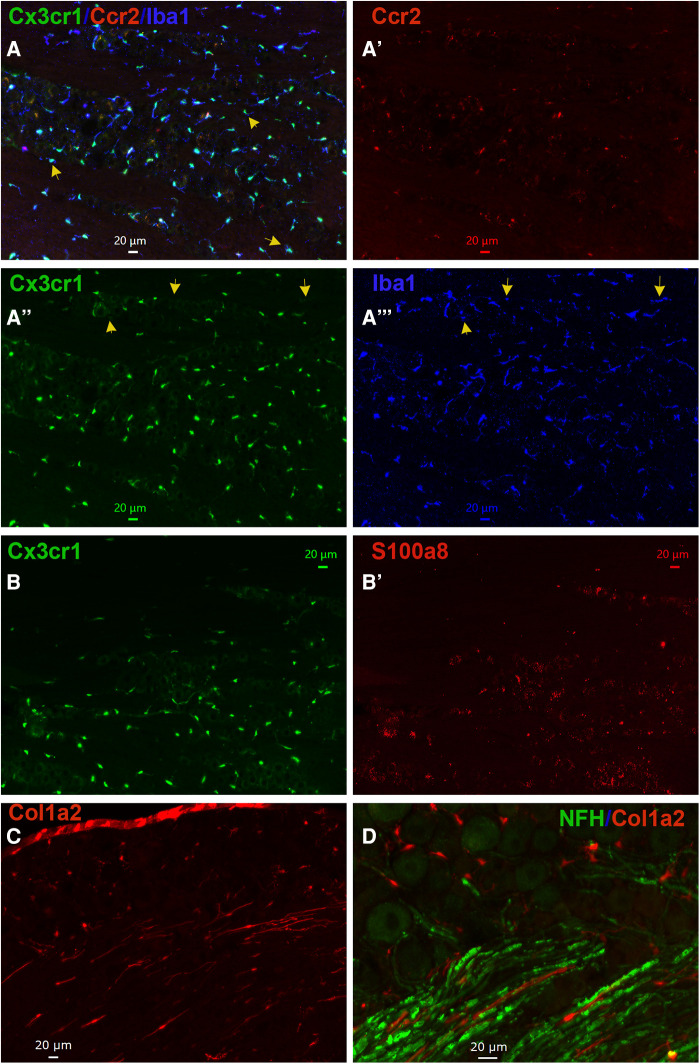
Representations of non-neuronal cells in TG. (**A**–**A**”’) Representative microphotographs of Ccr2^RFP^/Cx3cr1^GFP^ reporter mouse TG sections show relative expressions of macrophage markers Cx3cr1 (green), Ccr2 (red), and Iba1 (blue). The yellow arrows on panel A show Cx3cr1^+^/Iba1^+^/Ccr2^−^ macrophages in TG. The yellow arrows on panels (**A**”) and (**A**”’) show Cx3cr1^−^/Iba1^+^/Ccr2^−^ macrophages in TG. (**B–B**’) Representative microphotographs of Ccr2^RFP^/Cx3cr1^GFP^ reporter mouse TG sections show expression patterns of a macrophage marker Cx3cr1 (green) and a neutrophil/monocyte marker S100a8 (red). (**C**) A representative microphotograph of a Col1a2^cre^/Ai14^fl/^ reporter mouse TG sections shows the expression of a fibroblast marker Col1a2. (**D**) A representative microphotograph of a Col1a2^cre^/Ai14^fl/^ reporter mouse TG section shows relative expressions of a fibroblast marker Col1a2 and an A-fiber neuronal marker NFH. The scales are presented in each microphotograph.

**Figure 6:**
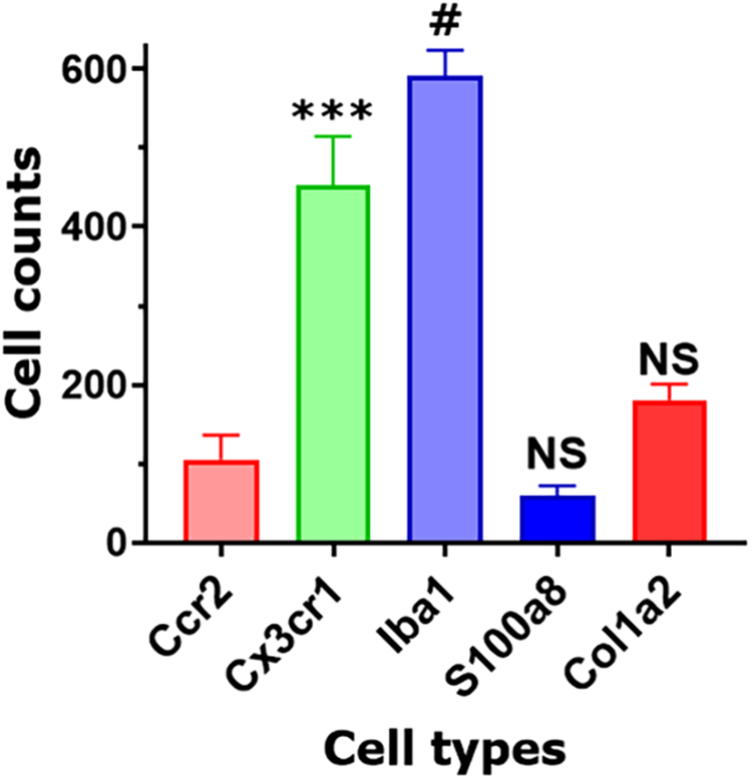
Non-neuronal cell count in TG sections. Counts of different types of non-neuronal cells in TG sections from Col1a2^cre^/Ai14^fl/^ and Ccr2^RFP^/Cx3cr1^GFP^ reporter mice. Every cell within a field of a microscope equipped with a ×20 objective was counted. Statistic is one-way ANOVA relative to the Ccr2 column (NS *p* > 0.05; ***p < 0.001; #*p* < 0.0001; *n* = 3).

Based on our scRNA-seq data, different types of Mph could be distinguished by labeling TG from Ccr2^RFP^/Cx3cr1^GFP^ reporter with Iba1 antibodies. In congruence with scRNA-seq data, three subsets of Mph—Cx3cr1^+^/Ccr2^−^/Iba1^+^ (Mph-1), Cx3cr1^+^/Ccr2^+^/Iba1^+^ (Mph-2), and Cx3cr1^−^/Ccr2^−^/Iba1^+^ (Mph-3)—could be distinguished (Figure 5A, Table 1). scRNA-seq revealed 633 and 618 cells in the Mph-1 and Mph-2 clusters, respectively, and 356 cells in the Mph-3 cluster (Figure 1B). Cell counting (see the Materials and Methods section) showed that there were substantially fewer Ccr2^+^ than Cx3cr1^+^ and Iba1^+^ cells in TG [one-way ANOVA; *F* (4, 10) = 42.48; *p* < 0.0001; *n* = 3; Figure 6].

A highly expressing Neu marker, s100a8 (Table 1), was detected using secondary antibodies with 647 fluorophores in a few TG cells (Figures 5B, 6). This result is surprising considering at least similar numbers of Mph and Neu in TG were revealed by scRNA-seq (Figures 1A,B). This indicated that in naïve WT mice, Neu is mainly located in the dura sheath covering the entire TG (see the next section) (41).

Fibroblasts were visualized by IHC on TG from Col1a2^cre^/Ai14^fl/−^ reporter mice. Fibroblasts with distinct shapes compared to immune and glial cells were noted among the neuronal cell bodies and myelinated nerve fibers in TG (Figures 5C,D). In summary, IHC can distinguish different types of TG Mph and visualize fibroblasts. IHC data also implied that Neu could be located in the dura sheath covering TG.

### Detection of TG immune cells by flow cytometry

**Figure 7:**
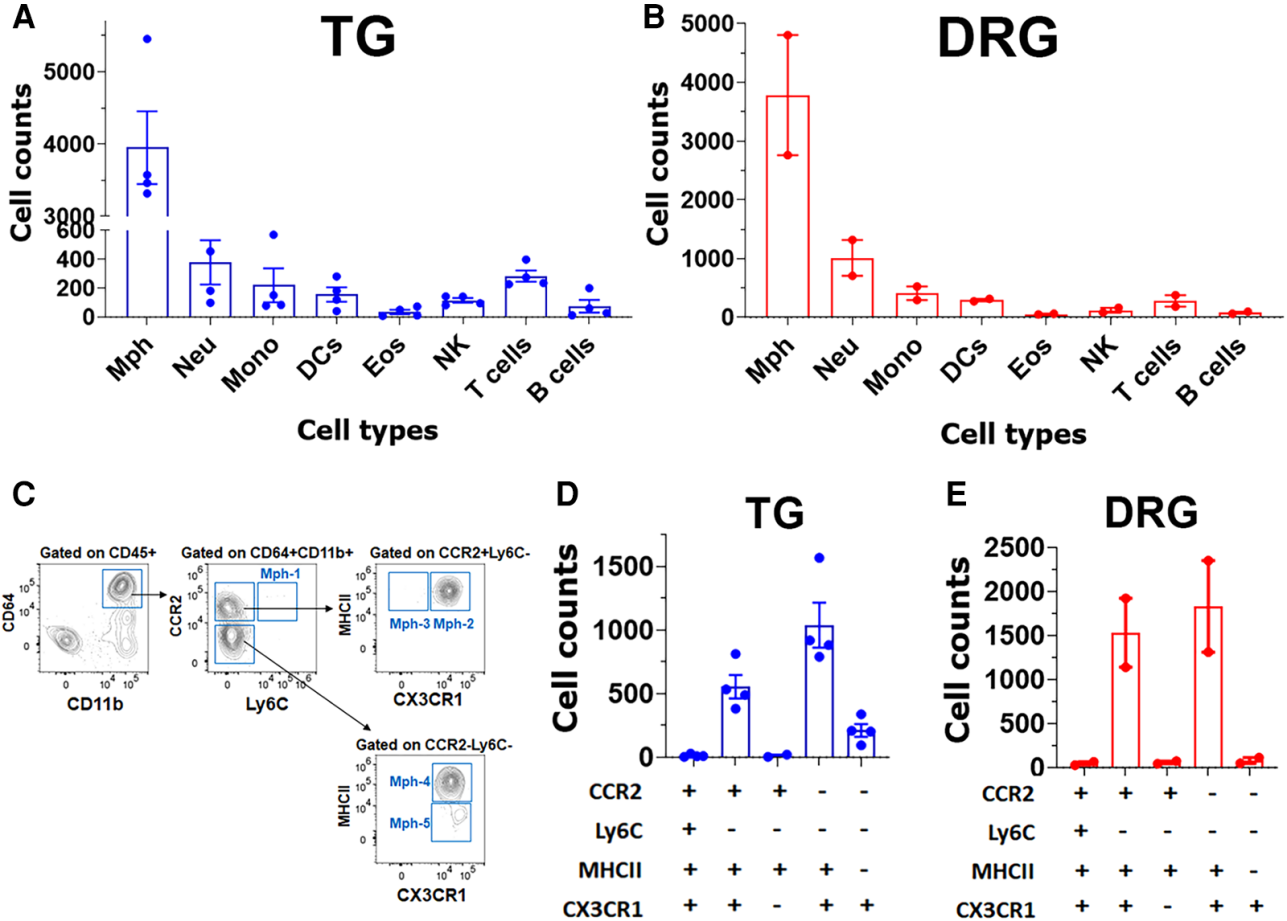
Immune cell profiles in TG and DRG isolations without surrounding dura. Flow cytometry count was normalized (by live cell numbers) immune cell counts in the TG (**A**) and DRG (**B**). WT male mouse ganglia were isolated without surrounding dura. Mph, macrophages; Neu, neutrophils; Mono, monocytes; DCs, dendritic cells; Eos, eosinophils; NK, natural killer cells; B cells; and T cells (*n* = 3–4). (**C**) Gating strategy to isolate a variety of macrophage types (Mph-1–Mph-5). Used markers are indicated at the *y*- and *x*-axis. (**D**,**E**) Normalized (by live cell numbers) cell counts for different types of macrophages (Mph-1–Mph-5) in TG (**D**) and DRG (**E**) without surrounding dura. Used markers are indicated beneath panels (**D**) and (**E**).

To further validate the findings of scRNA-seq on immune cells, we have performed flow cytometry on single-cell suspensions generated from male mouse TG. TG was isolated without or with surrounding dura (see Materials and Methods section). DRG was also isolated from some animals. The overall flow cytometry gating strategy is presented in Figure 2. TG and DRG without dura were dominated by Mph (Figures 7A,B). Neu and T cell levels are low in naïve WT mice (Figures 7A,B). It could be noted that Neu has a smaller sample size (*n* = 3) due to the failed actions of Neu (Ly6G) antibodies in one of the probes. Based on scRNA-seq and IHC results, we have designed a gating strategy to detect different types of Mph (Figure 7C). Neither TG nor DRG had Ly6C^+^/CCR2^+^ Mph-1, which could be classified as inflammatory Mph (Figures 7D,E). This Mph-1 is not present in TG and DRG from WT naïve male mice. TG and DRG also lacked Ly6C^−^/CCR2^+^/CX3CR1^−^/MHCII^+^ Mph-3 (Figures 7D,E). Both TG and DRG had Ly6C^−^/CCR2^+^/CX3CR1^+^/MHCII^+^ Mph-2 and Ly6C^−^/CCR2^−^/CX3CR1^+^/MHCII^+^ Mph-4 as dominant subsets (Figures 7D,E). This Mph-2 corresponds to Mph-2 (Ccr2) depicted in scRNA-seq (Figure 1B). Mph-4 detected by flow cytometry was Mph-1 (Cd163) depicted in scRNA-seq (Figure 1B). Finally, Ly6C^−^/CCR2^−^/CX3CR1^+^MHCII^−^ Mph-5 were present in TG, but not in DRG (Figures 7D,E). Flow cytometry-detected Mph-5 represents Mph-3 (Iba-1) clustered after scRNA-seq (Figure 1B). This Mph group is Iba-1^high^/Cx3cr1^low^.

**Figure 8:**
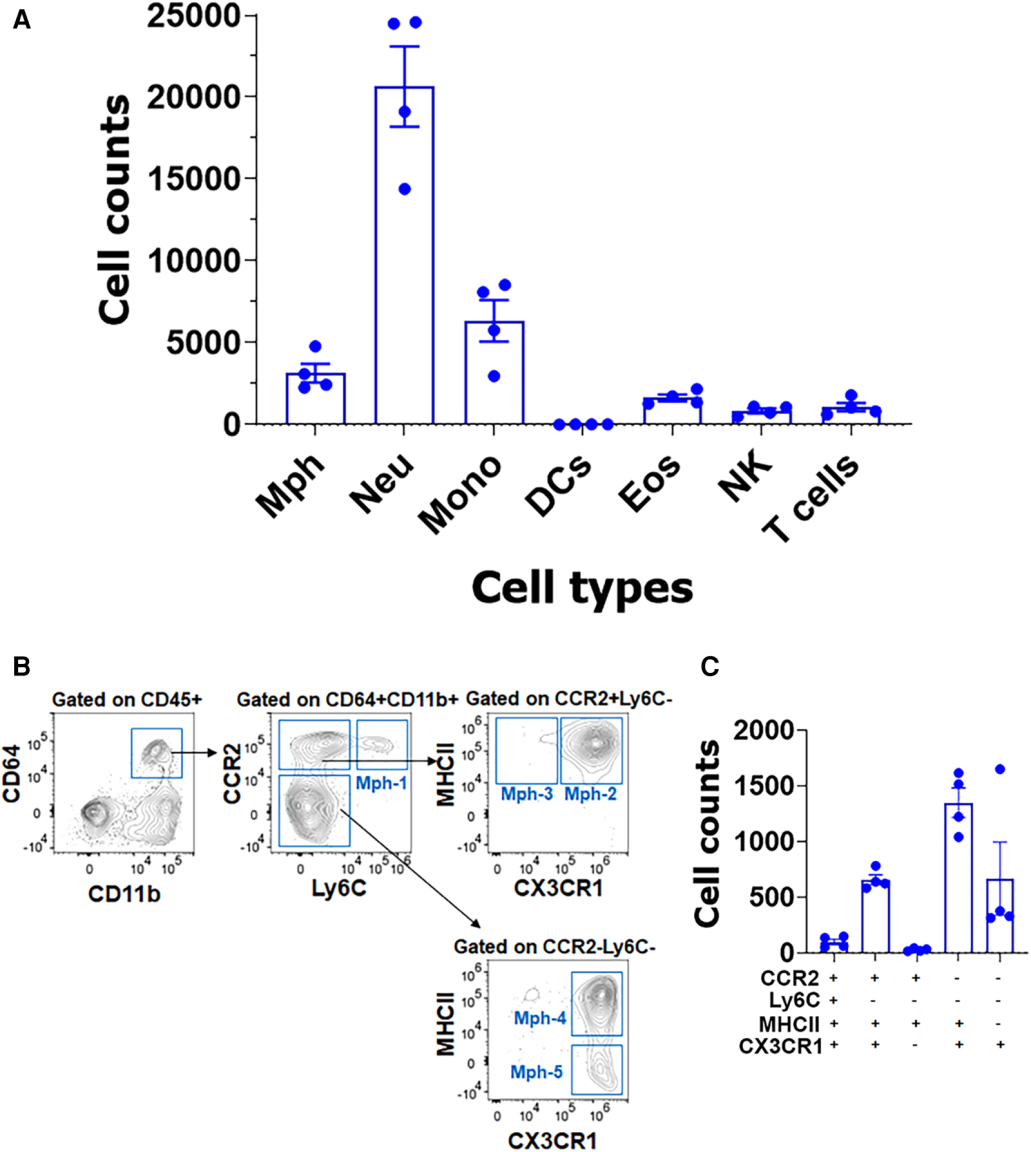
Immune cell profiles in TG isolations with surrounding dura. (**A**) Flow cytometry count was normalized (by live cell numbers) immune cell counts. TG from naïve Ccr2^RFP^/Cx3cr1^GFP^ reporter male mice was isolated with surrounding dura. Mph, macrophages; Neu, neutrophils; Mono, monocytes; DCs, dendritic cells; Eos, eosinophils; NK, natural killer cells; and T cells (*n* = 3–4). (**B**) Gating strategy to isolate a variety of macrophage types (Mph-1–Mph-5). Used markers are indicated at the *y*- and *x*-axis. (**C**) Normalized (by live cell numbers) cell counts for different macrophage types in TG with surrounding dura. Used markers are indicated below the *x*-axis.

Isolation of TG with surrounding dura, which is described in the Materials and Methods section, dramatically changed immune cell profiles (Figure 8A). Neu dominated the CD45^+^ cells (Figure 8A). In addition to Neu, the isolation of TG with dura led to an increase in other immune cells, especially monocytes (MO; Figure 8A). Interestingly, the isolation of TG with dura did not alter ganglion Mph and DC profiles (Figures 8B,C vs. Figures 7D,E). Thus, TG with dura had Ly6C^−^/CCR2^+^/CX3CR1^+^/MHCII^+^ Mph-2 and Ly6C^−^CCR2^−^/CX3CR1^+^/MHCII^+^ Mph-4 groups (Figures 8C). However, it appears that Mph with only Ly6C^−^CCR2^−^/CX3CR1^+^/MHCII^−^ Mph-5 were more present in TG with dura compared with TG without dura (Figure 8C vs. Figure 7E). This finding indicates that Iba-1^high^/Cx3cr1^low^ Mph are mainly in dura surrounding TG, but only a few in TG ganglia. Indeed, we counted a few of these Iba-1^high^/Cx3cr1^low^ Mph-3 in WT male mouse TG. Overall, the flow cytometry study validated the scRNA-seq data on the diversity of Mph and Neu in TG and showed that Neu was mainly presented in the dura surrounding TG from naïve WT mice.

### Comparison between TG immune cells and immune cells from published databases

We further leveraged the vast knowledge base of mouse immune cell types in our analysis of the identities of TG immune cells. To do so, we have compared TG immune cell transcriptional profiles to two major scRNA-seq datasets, referred to as the Ydens (42) and the Van Hove datasets (19). These studies reported transcriptional profiles of immune cells in the nervous system and compared them to peripheral myeloid cells, especially Mph (19). TG Mph and Neu had none-to-very low [<0.1 prediction score (PS)] matches with plasmacytoid DCs (pDCs, Siglech^+^/Ccr9^+^/Pacsin1^+^), conventional DCs that have been subdivided into the cDC1 (Flt3^+^/Irf8^hi^/Xcr1^+^) and cDC2 (Flt3^+^/Irf8^lo^/Cd209^+^) subsets, and migratory DCs (migDCs; Ccr7^+^/Nudt17^+^) (43, 44, 19) (Supplementary Figure S1). As expected, Mph and Neu had also none-to-very low (<0.1 PS) similarities with the adaptive immune system cells, such as NK (NK1.1^+^/TCRβ^−^), NKT, T (CD3^+^), B (CD22^+^), and ILC cells (Supplementary Figure S1) (19).

**Figure 9:**
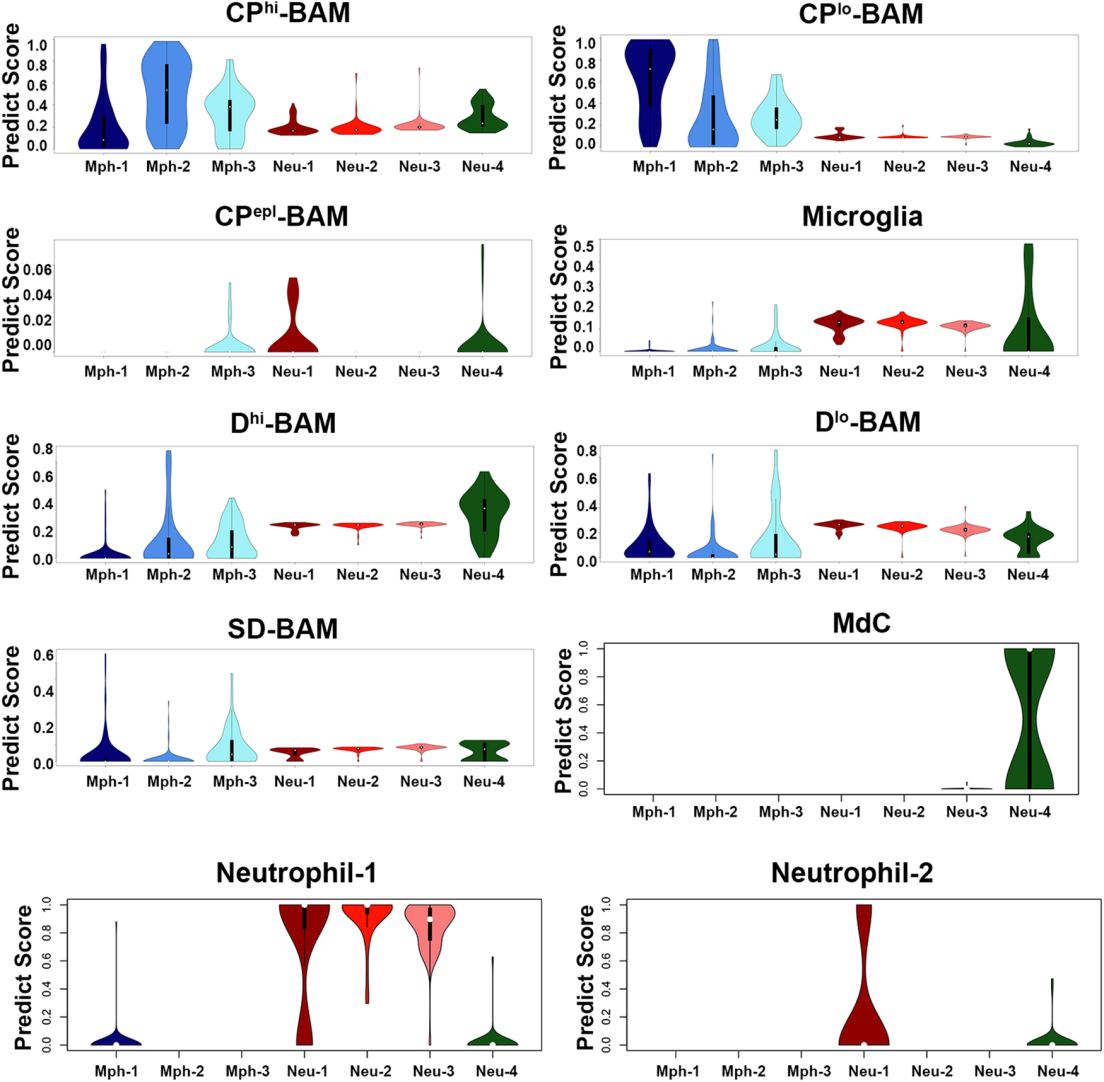
Comparison of mouse TG immune cell types with datasets. Prediction scores of our data against the Van Hoven dataset. The *y*-axis is the prediction score (aka probability score). The *x*-axis shows immune cell types of our dataset. The Van Hoven dataset cell-type nomenclature above each panel. Boxplots define the median. CP^hi^-BAM is choroid plexus high border-associated macrophages; CP^lo^-BAM is choroid plexus low border-associated macrophages; CP^epl^-BAM is choroid plexus-specific border-associated macrophages; D^hi^-BAM is dural high border-associated macrophages; D^lo^-BAM is dural low border-associated macrophages; SD-BAM is sub-dural border-associated macrophages; MdC is monocyte-derived cells.

Microglia, which was highly represented in the brain (19, 42), was distinct compared to TG Mph and Neu (Figure 9 and Table 2). TG Mph and Neu have also no match to both canonical and non-canonical monocytes (45). However, Neu-4 had substantial similarity to an intermediate monocyte/macrophage or monocyte/Neu populations [aka monocyte-derived cells (MdCs); Figure 9]. Moreover, unlike Neu-1, Neu-2, and Neu-3, Neu-4 did not exhibit transcriptional profiles of brain Neu (Figure 9). Neu-1, Neu-2, and Neu-3 belonged to the Neu-1 group, and poorly matched to the Neu-2 group (19) (Figure 9). However, the Neu-1 group was not divided into sub-groups as TG Neu (Figures 1A,B) (19).

**Table 2:** Markers of sciatic nerve macrophages in TG immune cells.

Gene ID	Mph-1	Mph-2	Mph-3	Neu	Others
Ccl12	6.1385	4.9514	2.4820	–	–
Cx3cr1	3.5415	2.5622	–	–	–
Fcrls	7.0536	3.6958	5.2903	–	–
Gas6	1.7846	–	1.9584	–	Fibro-2
Gpr34	1.4193	–	–	–	–
Mef2C	3.3218	2.1234	–	–	Endo
Sgce	–	–	–	–	–
Siglech	–	–	–	–	–
St3gal6	–	–	–	–	Endo
Tagap	–	–	–	–	–
Sema4d	–	–	–	–	–
Cxcl5	–	–	–	–	–
Trem2	2.3287	2.4142	1.7884	–	–
Hexb	4.2303	4.6987	3.0327	–	–
Adam19	–	–	–	–	–
Cbr2	1.4164	–	2.3256	–	–
Ccl8	–	–	–	–	–
Ccr2	–	1.0530	–	Neu-4	–
Cd209a	–	–	–	–	–
Cd209d	–	–	–	–	–
Cd36	–	–	–	–	–
Cd83	1.6209	3.4998	–	Neu-4	–
Clec10a	–	–	1.1288	–	–
Clec4n	1.0388	–	1.1084	–	–
Cxcl1	–	–	–	–	Fibro-1
Cxcl2	2.3639	9.7906	36.2917	Neu-2, Neu-4	–
Egfr	–	–	–	–	–
Enkur	–	–	–	–	–
Folr2	–	–	–	–	–
Foxred2	–	–	–	–	–
Fxyd2	–	–	–	–	–
H2–Aa	38.1446	51.9887	23.7860	Neu-4	Endo
H2–Ab1	41.0393	53.8268	23.1944	Neu-4	Endo
IL18rap	–	–	–	–	–
IL1rl1	–	–	–	–	–
Kmo	–	–	–	–	–
Mgl2	5.7038	4.8163	3.1959	–	–
Mmp9	–	–	–	–	–
Mpz	1.2528	–	–	Neu-3	Glia, Fibro-2
Msr1	–	–	–	–	–
Myo5a	–	–	–	–	–
Pla2g2d	–	–	–	–	–
Polg2	–	–	–	–	–
Ptgs2	–	–	–	–	–
Retnla	–	–	–	–	–
Qpct	–	–	–	–	–
Selm	–	–	–	–	Glia, Fibro-1, Fibro-2
Slamf7	–	–	–	–	–
Thap6	NA	NA	NA	NA	NA
Timd4	–	–	–	–	–
Tmod1	–	–	–	–	–
Tlr8	–	–	–	–	–
Tslp	–	–	–	–	–
Ugt8a	–	–	–	–	–
Xist	–	–	–	–	–
Adora3	–	–	–	–	–
Cd34	–	–	–	–	Fibro-1, Endo
Crybb1	–	–	–	–	–
Csmd3	–	–	–	–	–
Ecscr	–	–	–	–	Endo
Fscn1	–	–	–	–	–
Grp56/Adgrg1	–	–	–	–	–
H2–Oa	–	–	–	–	–
Kcnd1	–	–	–	–	–
Lag3	–	–	–	–	–
Nav3	–	–	–	–	–
Sall1	–	–	–	–	–
Sall3	–	–	–	–	–
Scl24a3	NA	NA	NA	NA	NA
Scl2a5	NA	NA	NA	NA	NA
Tmc7	–	–	–	–	–
Tmem119	–	–	–	–	–

The blue font for gene ID shows the genes specific for both microglial and sciatic macrophages; the brown font for gene ID shows the genes specific for sciatic macrophages; the green font for gene ID shows the genes specific for microglia; the – sign means an expression below 1; *NA* marks non-sequenced genes; the *Others* column shows the expression of genes in other non-neuronal TG cells.

Mph in the brain tissues were differentiated from microglia based on a high expression of prototypical Mph genes, such as *Adgre1* (aka F4/80) and *Fcgr1* (aka CD64) (19, 42). Mph in the central nervous system (CNS) were detected in the whole-brain samples and especially border regions, such as the dura mater, choroid plexus (CP), and sub-dura meninges (19). These Mph in CNS are called BAMs (46). Transcriptional profiles and morphology of BAMs are drastically different from peritoneal and alveolar Mph and Kupffer cells (19, 42). Thus, TG Mphs have a similar shape to BAMs [Figure 1A vs. Figure 4A from (19)]. Transcriptional profiles of immune cells from distinct border regions revealed six major BAM subsets (19). The comparison of TG Mph with these BAM subsets showed that the Mph-1 group has the highest match to CP-low BAMs (CP^lo^-BAMs); Mph-2 is similar to CP-high BAMs (CP^hi^-BAMs), while Mph-3 is distinct and has <0.5 PS to the reported BAMs (19) (Figure 9). TG Mph had minimal similarities with two dural BAMs (i.e., D^lo^-BAMs and D^hi^-BAMs), sub-dura BAMs (SD-BAMs), and small CP group (CP^epi^-BAMs) (19) (Figure 9). Moreover, the analysis of gene marker expressions showed that TG Mph was also different from sciatic Mph (Table 2), which were presented as a distinct group (42).

**Figure 10:**
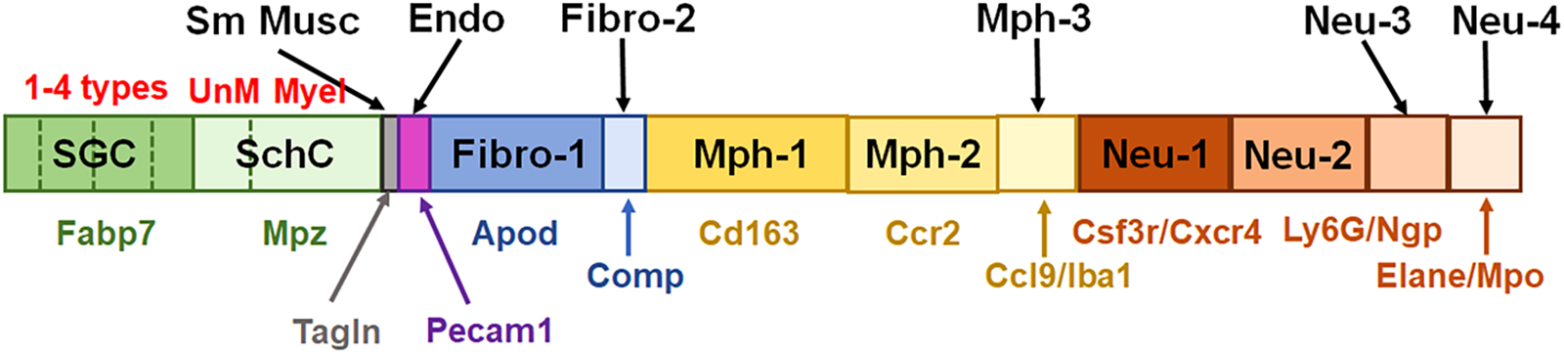
Schematic for representation of non-neuronal cells in TG. This schematic summarizes published and generated here data. Satellite glial cells (SGC) are represented by four different subtypes. SchC—Schwann cells are represented by two subtypes: unmyelinated (UnM) and myelinated (Myel). Sm Musc—vascular smooth muscle cells. Endo—vascular endothelial cells. Fibro—fibroblasts are divided into two sub-groups, i.e., Fibro-1 and Fibro-2. Mph—macrophages are divided into three sub-groups: Mph-1, Mph-2, and Mph-3. Neu—neutrophils are divided into four sub-groups: Neu-1, Neu-2, Neu-3, and Neu-4. Gene markers for each TG non-neuronal cell type are indicated underneath the bars with the same color theme as the bars.

In summary, multiple independent repetitions of scRNA-seq studies positively contribute to the generation of accurate single-cell profiles for tissues at naïve and pathological conditions. Accordingly, we performed two different non-neuronal cell isolations and two separate replicates (reported separately in Figures 1, 3, 4) of scRNA-seq to generate transcriptional profiles for non-neuronal TG cells. Then, based on marker expression profiles, we integrated previously published and our data to generate a schematic depicted in Figure 10. One of the distinct findings of the presented study is the identification of multiple Mph and Neu in TG samples. Some of them match to reported BAMs from the CP, but some of them are distinct for TG.

## Discussion

The plasticity of non-neuronal cells in TG and DRG during a variety of acute and chronic pain conditions was reported in multiple publications. It was suggested that this plasticity of glial (1–4) and immune cells (5–8) has essential contributions to the progression of pain in many diseased states. It is presumed that transcriptional changes in sensory ganglion non-neuronal cells could eventually lead to the production of a plethora of mediators. It has been shown that these mediators can sensitize sensory neurons (5, 2, 3) via direct contact of non-neuronal cells with sensory neurons (28) and/or by regulating a plethora of neuronal channels, which could result in a change in neuronal excitability and gating properties (7, 9, 4).

This essential role of non-neuronal ganglion cells in the regulation of sensory neuronal excitability and the development of pain conditions elevated information on transcriptional profiles for non-neuronal sensory ganglion cells to a critically important level. Such knowledge could be used to study the interactomic network between sensory neurons and non-neuronal cells (10). This dataset could also be a baseline in the investigation of TG non-neuronal cell plasticity in different pain models for the head and neck regions. RNA-seq on the single-cell level was employed in several publications to gain this important information and knowledge on transcriptional profiling of non-neuronal cells in mouse TG (13, 32, 14, 15). The results of these independent studies have substantial overlap but show some differences. These differences are unavoidable and could be dictated by nucleus/cell isolation approaches, the number of sequenced cells, sequencing depth, and clustering analysis (11–13, 32, 14). Thus, multiple independent repetitions of scRNA-seq positively contribute to the generation of accurate single-cell profiles for tissues at naïve and pathological conditions. Accordingly, we integrated previously published and our data (Figure 9).

There are several types of glial cells in TG (Figure 1). Schwann cells could be assigned to one of two categories, i.e., myelinated and non-myelinated (14, 15). SCG could also be divided into four subsets, i.e., general resident, sensory, IEG, and immune responsive (32). *Fabp7* is the specific marker for all SGC sub-groups and Mpz for all Schwann cell sub-groups. Suggested *Apoe* as an SGC marker is expressed at high levels in Mph (Table 1) (14, 15). *Plp1* is also not suitable as an SGC marker, because it is present at an equal level in Schwann cells and SGC (Table 1) (28, 29). Moreover, low-to-moderate levels of expression of glial markers have been reported in sensory neurons (31, 13, 14). Overall, we could not find viable markers to generate SGC or Schwann cell-specific reporter mice that could be used for activation, inhibitions, and ablations of these glial cell types.

Previous reports showed that among the TG non-neuronal cells, there were small numbers of vascular smooth muscle, endothelial cells, and two types of fibroblasts (14, 15) (Figure 1). Our scRNA-seq data concur with this finding (Figure 1). We suggest *Apod* as a gene marker for one group of fibroblasts and *Comp* for another group (Figure 1 and Table 1). *Dcn* could be another good marker choice for the Fibro-1 group, since *Apod* is expressed on substantial levels in sensory neurons (31, 13, 14). However, *Mgp* as a proposed marker for Fibro-2 is presented on significant levels in other TG non-neuronal cells (Table 1). In addition, it appears that the most appropriate marker to manipulate all fibroblasts is Col1a2. Our IHC data have shown that fibroblasts are among the sensory neuronal cell bodies in TG and nerve fibers.

The importance of sensory ganglion immune cells in the regulation of the development of pain conditions is well documented in many reports (see Introduction section). ScRNA and snRNA-seq showed only one immune cell cluster in TG (13–15). Interestingly, DRG contained Mph, Neu, and B cell clusters (12). Our data indicate that there are at least seven immune cell clusters in TG (Figure 10 and Table 1). IHC and flow cytometry confirmed the presence of three types of residential Mph (Cd64^+^) in TG, which could be recognized by preferential expression of Cd163, Ccr2, and Iba1 markers (Figures 4, 5 and Table 1) and separated by flow cytometry (Figures 7E, 8C). Interestingly, Cd163 is a marker for M2 Mph. Moreover, two recognized M2 markers, i.e., Cd206/Mrc1 and Il10rb, are also expressed on Mph-1 (see the Supplementary Material). Further analysis using the label transfer method demonstrated that Mph-1 is similar to CP^lo^-BAMs. Mph-2 has the highest prediction score with CP^hi^ BAMs, while Mph-3 is distinct (19). Flow cytometry data indicate that Mph-3 (Iba1^hi^/Cx3cr1^lo^) is mainly present in the dura surrounding TG. TG Mph are quite dissimilar to the dura, the sub-dura, a CP group CP^epi^ BAMs, and the microglia. The gating strategy described here is different from the one used for the isolation of BAMs from the CP, dura, and sub-dura. Thus, we have relied on the differential expression of MHCII, Ly6C, Ccr2, and Cx3cr1 (Figure 7), while Van Hove et al. used differential expression (low, intermediate, high) of P2r7, Clec12a, Folr2, and Nrp1 to separate the BAM subsets (19).

scRNA-seq data clustering revealed four types of Neu (s100a8^+^), which are either Ly6G^+^ or Ly6G^−^. Surprisingly, s100a8 antibodies, which were used in multiple publications to label Neu in several tissues, did not show a strong signal within naïve mouse TG. Neu were mainly located in the perineuronal/dura sheath surrounding DRG but infiltrated DRG during myalgia (41). Similarly, flow cytometry revealed plenty of Neu in TG isolation with surrounding dura (Figure 8A). Ly6G^+^ Neu were clustered into two groups, but we could not find specific markers distinguishing the Neu-2 sub-type from the Neu-3 sub-type (Table 1). Ly6G^−^ Neu could be readily differentiated into two groups using *Csf3r* or *Cxcr4* for Neu-1 and *Elane*, *Mpo*, or *Pycard* for Neu-4 (Figure 4, Table 1). The label transfer analysis showed that Neu-1, Neu-2, and Neu-3 were similar to the brain Neu-1 group, whereas Neu-4 had a resemblance to monocyte-derived cells.

In conclusion, comprehensive transcriptional profile data of TG non-neuronal and especially immune cells generated by our and several independent studies establish a foundation for detailed studies on the regulation of a variety of pain conditions by these cell sub-types. The detailed transcriptional profiles for each group of cells and independent replicates generated in this study are provided as Supplementary Material. Thus, as outlined here, information could allow monitoring of the plasticity of different types of TG myeloid cells, which are unique in their transcriptional profiles and belong to the BAM and Neu classes; generating novel tools (mouse lines) for selective manipulations of particular sub-types on TG non-neuronal and immune cells; and evaluating of cell plasticity during pain conditions. This, in turn, could address understudied areas of pain research by building the molecular basis for the mechanisms controlling the chronicity of a variety of pain conditions for the head and neck regions.

## Data Availability

Single-cell RNA-seq data has been deposited and has GEO Accession GSE240432. Supplementary excel files show the mean gene readings for each cluster. Supplementary files are *“Non-neuronal TG clusters (1^st^ run, rev).xlsx” and “Non-neuronal TG clusters (2^nd^ run, rev).xlsx”*.

## Supplementary material

Supplementary Figure S1 Comparison of mouse TG immune cell types with datasets. Prediction scores of our data against the Van Hoven dataset. Y-axis is prediction score (aka Probability Score). X-axis shows immune cell types of our dataset. The Van Hoven dataset cell type nomenclature above each panel. Boxplots define the median. cDC-1 is conventional dendritic cells type-1; cDC-2 is dendritic cells type-2; pDC-1 is plasmacytoid dendritic cells; migDC is migratory dendritic cells; NK cells is natural killer cells; NKT cells is natural killer T cells; ILC is innate lymphoid cells.
