# Supplementary figures and images for: Transcriptional profiles of non-neuronal and immune cells in mouse trigeminal ganglia
Source: Front Pain Res (Lausanne). 2023 Oct 31;4:1274811. doi: 10.3389/fpain.2023.1274811 (PMC10644122; doi:10.3389/fpain.2023.1274811)

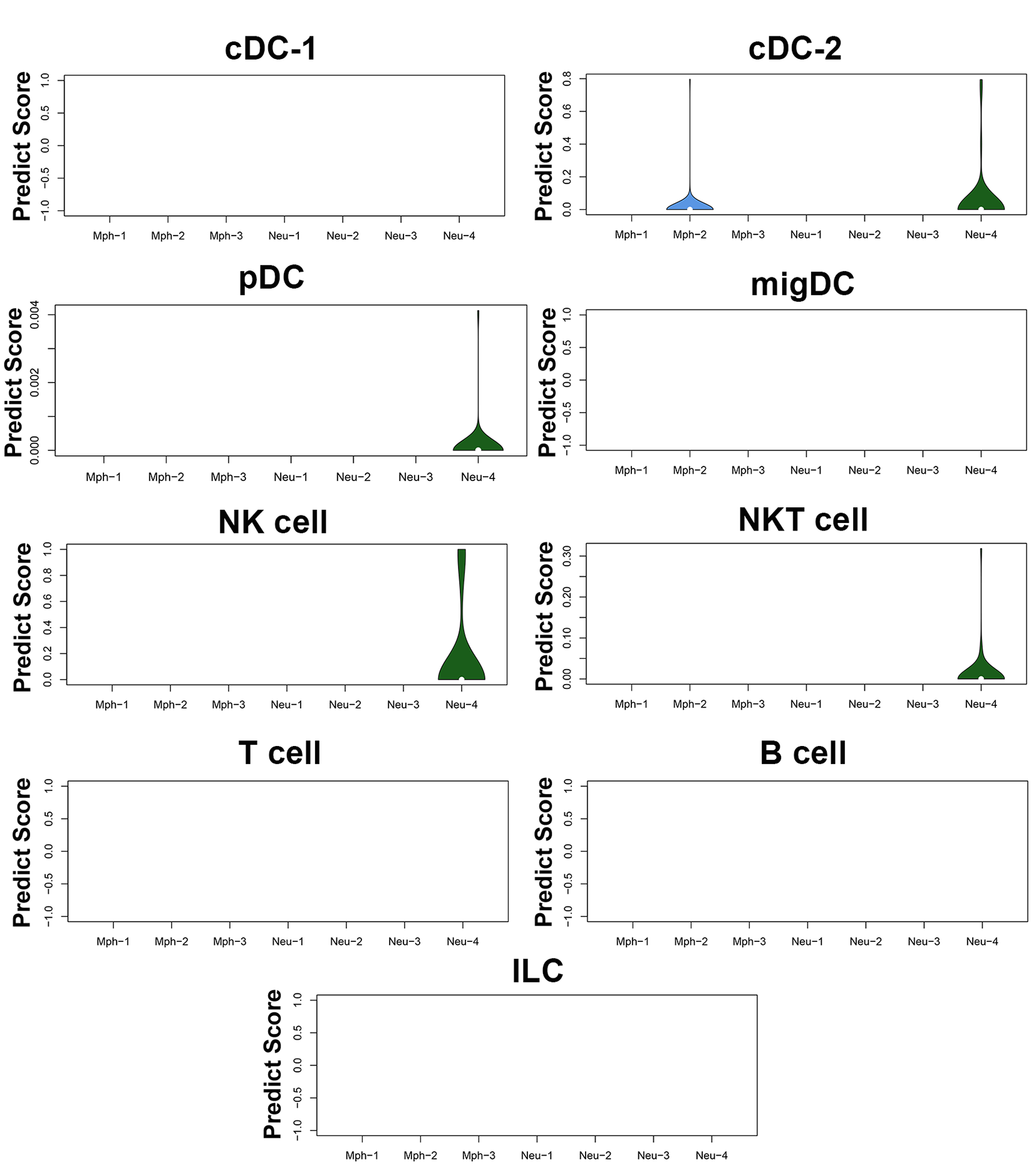

Supplement: Supplementary Figure S1 — Comparison of mouse TG immune cell types with datasets. Prediction scores of our data against the Van Hoven dataset. Y-axis is prediction score (aka Probability Score). X-axis shows immune cell types of our dataset. The Van Hoven dataset cell type nomenclature above each panel. Boxplots define the median. cDC-1 is conventional dendritic cells type-1; cDC-2 is dendritic cells type-2; pDC-1 is plasmacytoid dendritic cells; migDC is migratory dendritic cells; NK cells is natural killer cells; NKT cells is natural killer T cells; ILC is innate lymphoid cells. [file Image1.tif]
